# Supplementary material for: Tailoring Hot-Carrier Distributions of Plasmonic Nanostructures through Surface Alloying
Source: ACS Nano. 2024 Feb 16;18(8):6398–405. doi: 10.1021/acsnano.3c11418 (PMC10906084; doi:10.1021/acsnano.3c11418)
Supplement: Supplementary file 1 — nn3c11418_si_001.pdf [file nn3c11418_si_001.pdf]

Supporting Information

Tailoring hot-carrier distributions of plasmonic nanostructures through surface alloying

Jakub Fojt[1], Tuomas P. Rossi[2], Priyank V. Kumar[3], and Paul Erhart[1]

[1] *Department of Physics, Chalmers University of Technology, SE-412 96 Gothenburg, Sweden*
[2] *Department of Applied Physics, Aalto University, FI-00076 Aalto, Finland*
[3] *School of Chemical Engineering, The University of New South Wales, 2052 Sydney, NSW, Australia*

Contents

Supplementary Figures **2**
S1. Geometry of surface alloys 2
S2. Normalized number of electrons and holes generated at the surface for different surface alloy compositions 2
S3. Spatially resolved number of generated electrons and holes for various full-layer compositions 3
S4. Spatially resolved HC distributions for various full-layer compositions 4
S5. Carrier distributions at the top surface of alloyed and unalloyed NPs 5
S6. Spectra of Pt-surface alloyed Ag NPs of different sizes and shapes 6
S7. Hot-carrier distributions in the surface layer for Pt-surface alloyed Ag NPs of different sizes and shapes 7
S8. Total number of generated carriers in the surface layer for Pt-surface alloyed Ag NPs of different sizes and shapes 7
S9. Number of HCs at the surface of the core-crown Ag–Pt NP for different surface alloy compositions and alloyants 8
S10. Number of electrons and holes generated at the surface of alloyed and unalloyed NPs . . 8

Supplementary Figures

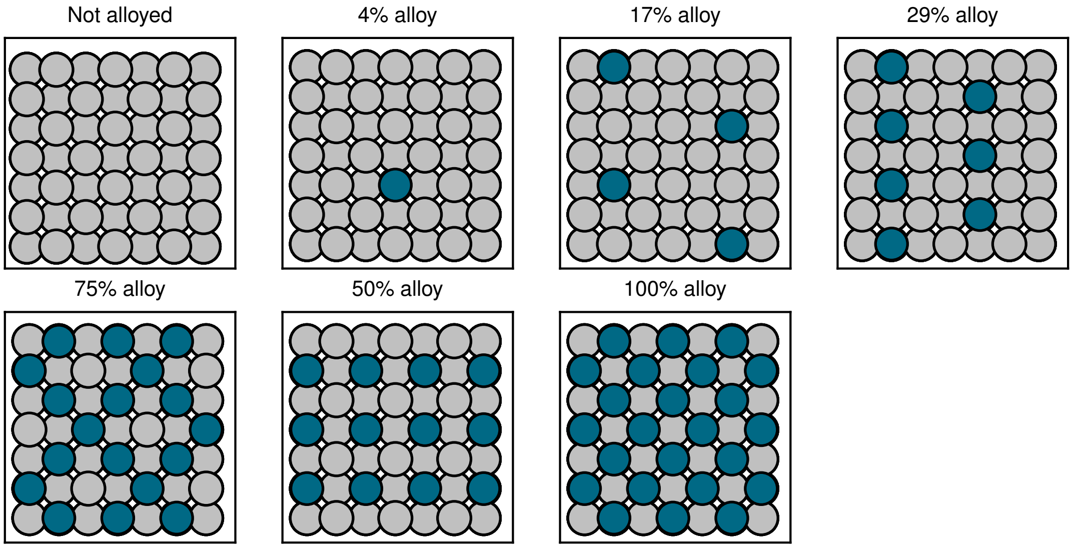

Figure S1: **Geometry of surface alloys.** View of the alloyed surface from the top.

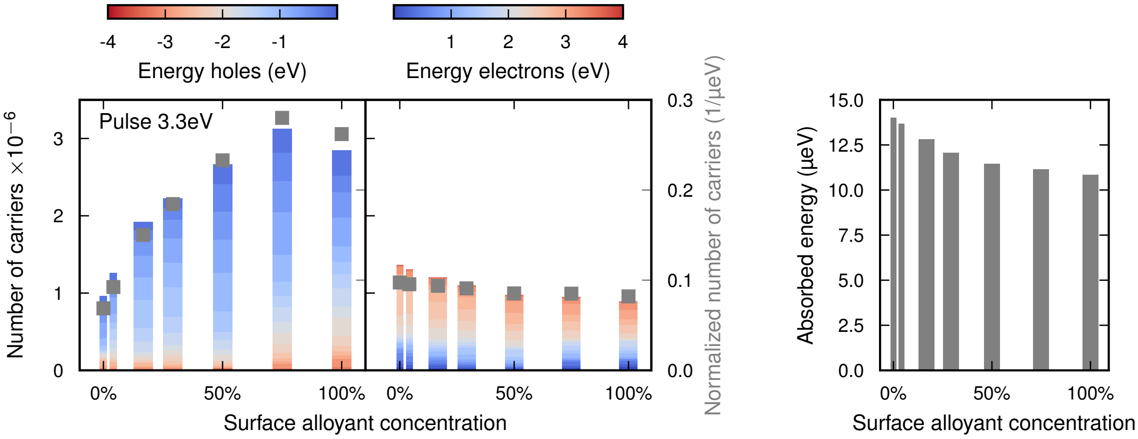

Figure S2: **Normalized number of electrons and holes generated at the surface for different surface alloy compositions.** The total number of HCs in the surface (left panel, bars), the total energy absorbed by the NP (right panel), and the former normalized by the latter (left panel, squares) are shown. The alloyant is Pt and the system was excited with a laser at the LSP peak (3.3 eV).

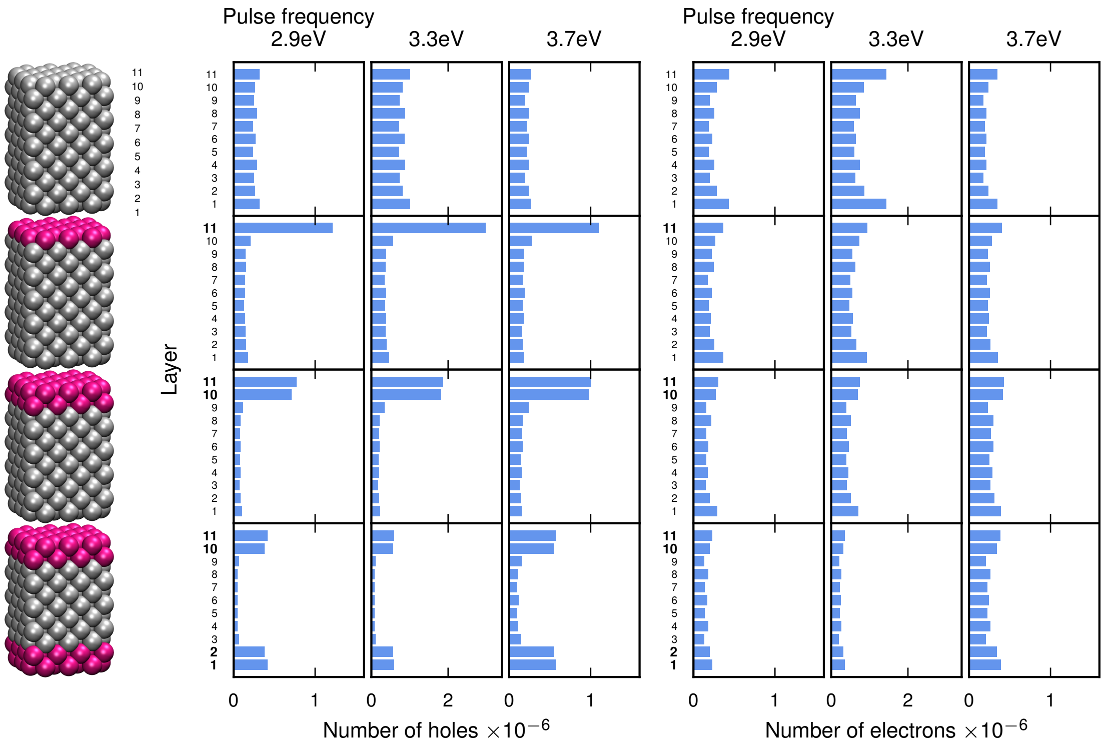

Figure S3: **Spatially resolved number of generated electrons and holes for various full-layer compositions.** Substitutions of Pt have been done in no layers, one top layer, two top layers, and two bottom and two top layers, as indicated by the highlights in the atomic structures. The non-alloyed NP has a relatively uniform spatial distribution of holes, and an electron distribution that is higher near the edges. Alloyed NPs have large numbers of holes localized to the alloy layers, while such a localization effect is lacking for electrons. Note the different scales for different pulse frequencies.

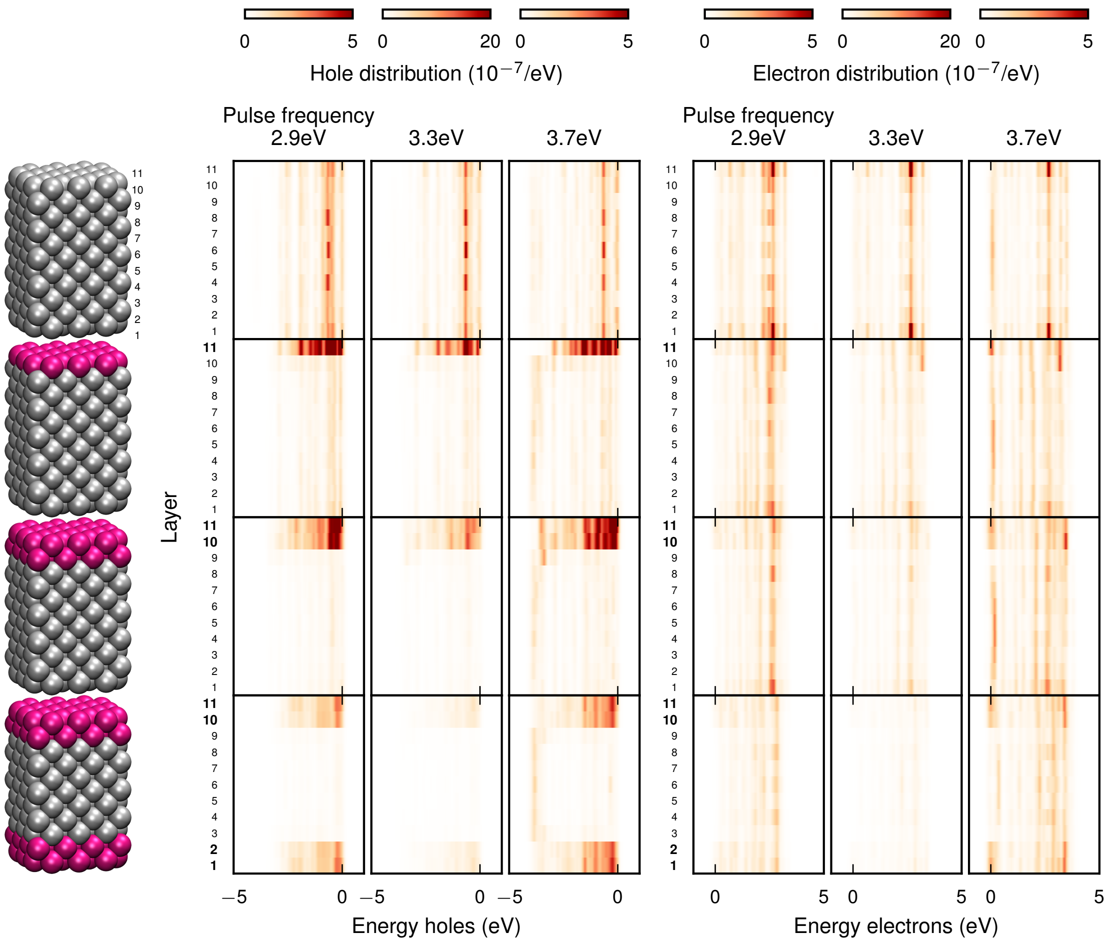

Figure S4: **Spatially resolved HC distributions for various full-layer compositions.** Substitutions of Pt have been done in no layers, one top layer, two top layers, and two bottom and two top layers, as indicated by the highlights in the atomic structures. For alloyed NPs, holes corresponding to the d-states of Pt are localized to the Pt layers, at energies between −2 and 0 eV. Such a localization effect is lacking for the electrons. For large pulse frequencies (3.7 eV), transitions from the d-band of Ag (−3.8 eV, holes) to the Fermi level (electrons) are possible, which is seen in the Ag layers. Note the different scales for different pulse frequencies.

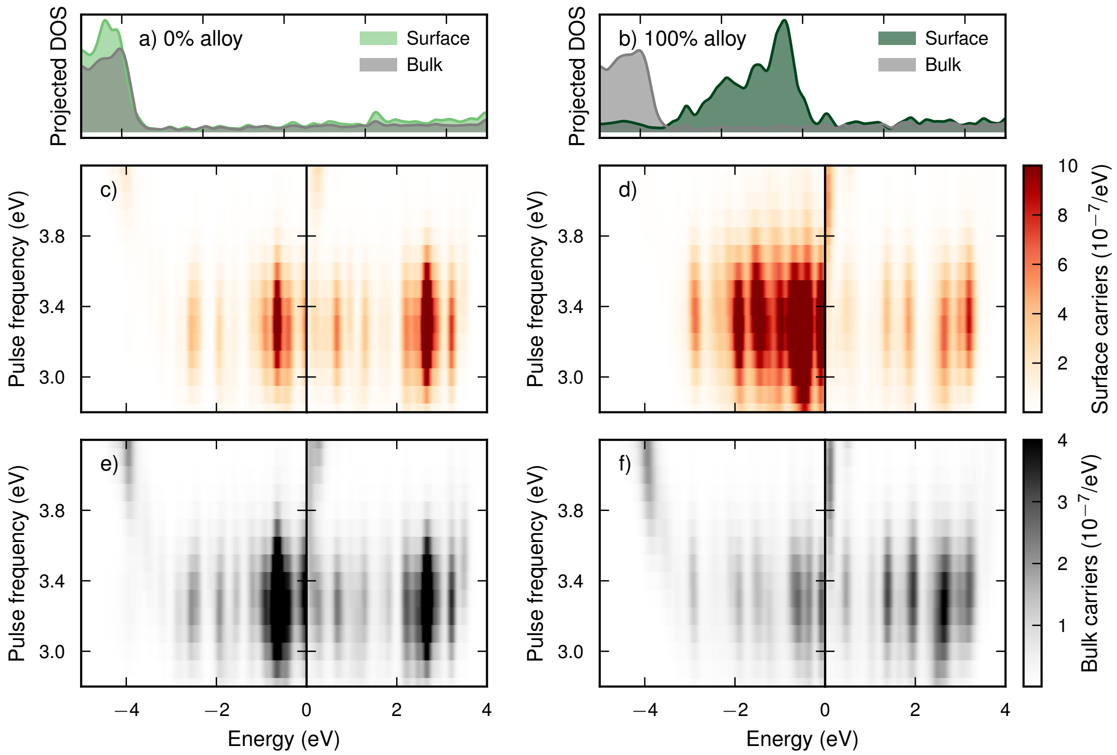

Figure S5: **Carrier distributions at the top surface of alloyed and unalloyed NPs.** (a-b) Projected densities of state, (c-d) surface and (e-f) bulk carrier distributions, for different exciting pulse frequencies. The alloyant is Pt. Note the different scales between surface and bulk.

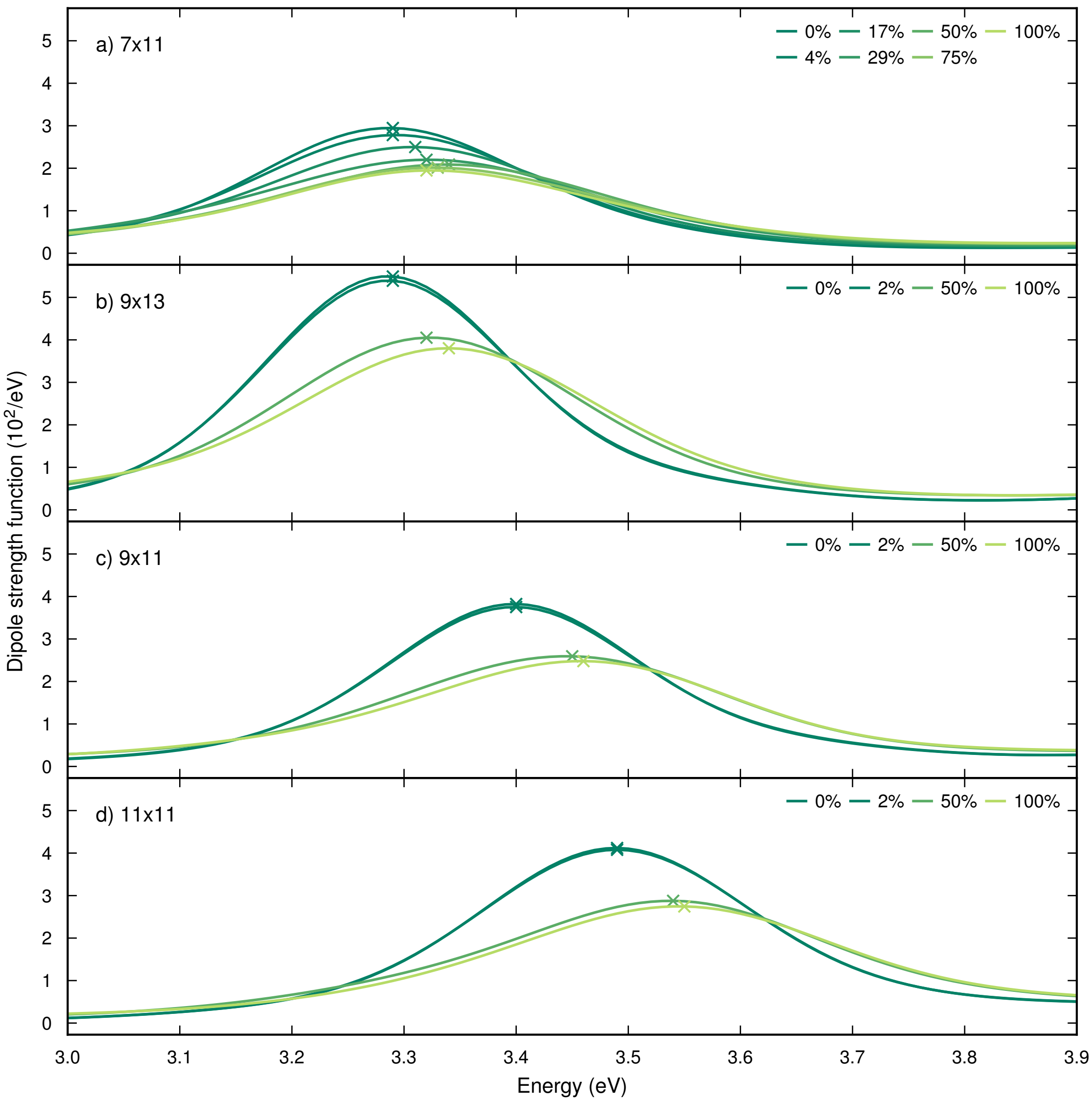

Figure S6: **Spectra of Pt-surface alloyed Ag NPs of different sizes and shapes.** All NP shapes exhibit the same trend, where the LSP resonance blue-shifts and broadens with increasing alloyant concentration. The subpanel labels denote respectively the number of atomic layers on the short axes and on the long axis (the latter being the polarization direction) of the NPs. The different lines show different Pt surface concentrations as indicated in the legends.

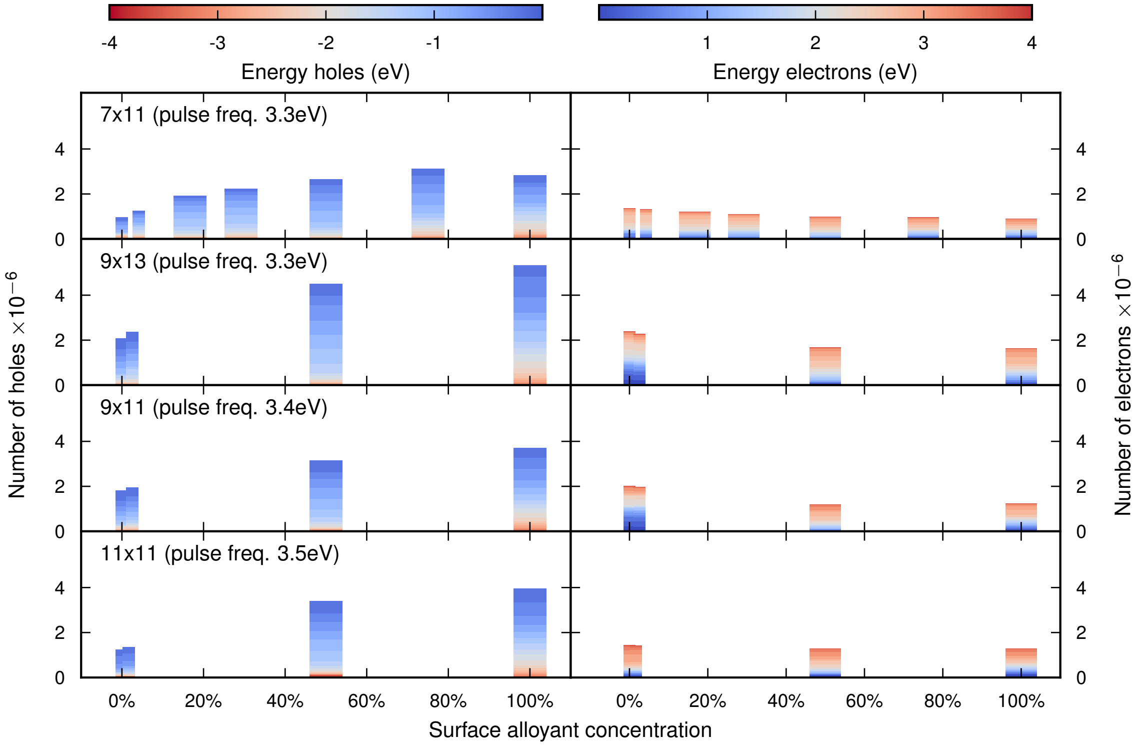

Figure S7: **Hot-carrier distributions in the surface layer for Pt-surface alloyed Ag NPs of different sizes and shapes.** All NP shapes exhibit the same trend, where the number of holes in the surface layer increases greatly, and the number of electrons in the same layer decreases slightly, with increasing surface alloyant concentration. The increase in the number of holes is greater between 0 and 50 % than between 50 and 100 %. The subpanel labels denote respectively the number of atomic layers on the short axes and on the long axis (the latter being the polarization direction) of the NPs, and the pulse frequency used (chosen to be close to the LSP resonance frequency).

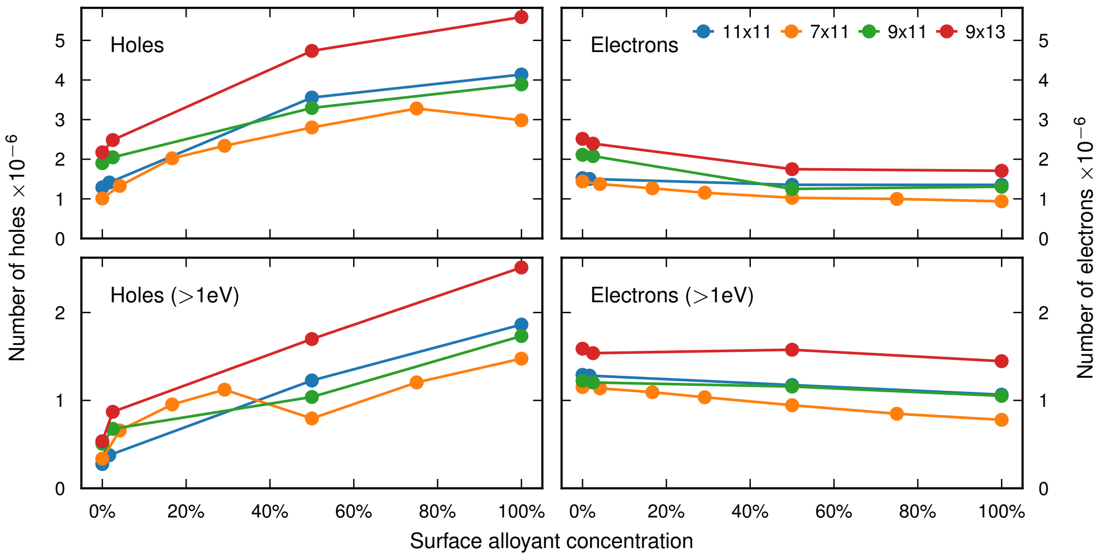

Figure S8: **Total number of generated carriers in the surface layer for Pt-surface alloyed Ag NPs of different sizes and shapes.** Alternative visualization of the data in Fig. S7.

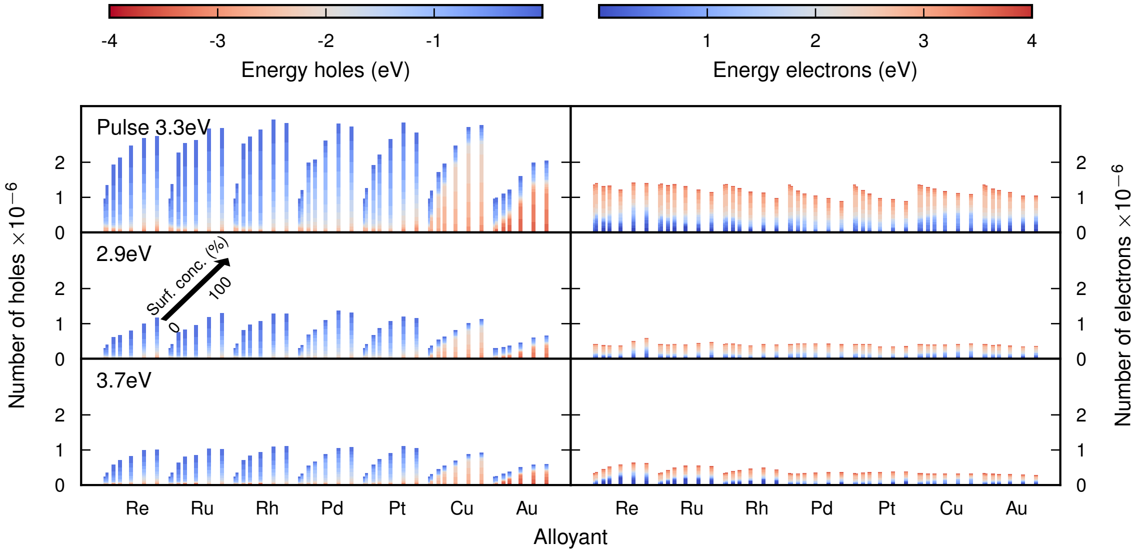

Figure S9: **Number of HCs at the surface of the core-crown Ag–Pt NP for different surface alloy compositions and alloyants.**

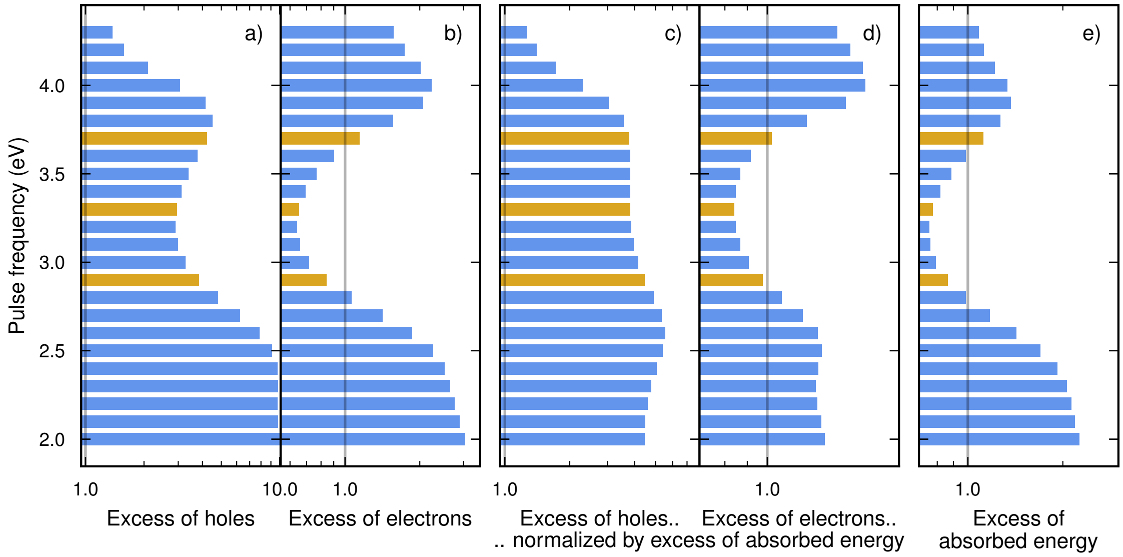

Figure S10: **Number of electrons and holes generated at the surface of alloyed and unalloyed NPs.** (a-b) The number of holes and electrons in the surface layer of the 100 % Pt surface alloy divided by the number of holes and electrons in the same surface of the non-alloyed NP. (b-c) The same quantities as in (a-b), scaled by the quantity in (e). (e) The amount of energy absorbed in the entire alloyed NP, divided by the amount of energy absorbed in the non-alloyed NP.

For pulse frequencies between 2.9 and 3.6 eV, the amount of energy absorbed in the alloyed NP is lower (e), due to lower oscillator strength. For the same frequencies, the number of electrons excited in the surface layer is decreased in the alloyed NP (b), even when compensating for the lower amount of energy absorbed (d).
